# Consequences of community water fluoridation cessation for Medicaid-eligible children and adolescents in Juneau, Alaska

**DOI:** 10.1186/s12903-018-0684-2

**Published:** 2018-12-13

**Authors:** Jennifer Meyer, Vasileios Margaritis, Aaron Mendelsohn

**Affiliations:** 1 Health Sciences, University of Alaska Anchorage, College of Health, 3211 Providence Drive, Anchorage, AK 99508 USA; 2 Public Health Programs, School of Health Sciences, College of Health Sciences, Walden University, 100 Washington Ave. South, Suite 900, Minneapolis, MN 55401 USA

**Keywords:** Dental caries, Water fluoridation, Alaska

## Abstract

**Background:**

The general aim of this research was to determine whether cessation of community water fluoridation (CWF) increased oral health disparities, as measured by dental caries procedures and restoration costs for children and adolescents.

**Methods:**

The analysis was based on all Medicaid dental claims records of 0- to 18-year-old patients residing in zip code 99801 (Juneau, Alaska) during an optimal CWF year (2003, *n* = 853) compared to all claims for the same age group from 2012 (*n* = 1052), five years after cessation of CWF. A bivariate analysis (Mann-Whitney U test) of the mean number of caries procedures performed per client was conducted in the study groups under both independent CWF conditions. Furthermore, logistic regression was performed using the dependent variables of caries procedures and the cost of caries-related procedures, with adjustments for CWF group, gender, and race.

**Results:**

The statistically significant results included a higher mean number of caries-related procedures among 0- to 18-year-old and < 7-year-old patients in the suboptimal CWF group (2.35 vs. 2.02, *p* < 0.001; 2.68 vs. 2.01, *p* = 0.004, respectively). The mean caries-related treatment costs per patient were also significantly higher for all age groups, ranging from a 28 to 111% increase among the suboptimal CWF cohorts after adjusting for inflation. The binary logistic regression analysis results indicated a protective effect of optimal CWF for the 0- to 18-year-old and < 7-year-old age groups (OR = 0.748, 95% CI [0.62, 0.90], *p* = 0.002; OR = 0.699, 95% CI [0.52, 0.95], *p* = 0.02, respectively). Additionally, the age group that underwent the most dental caries procedures and incurred the highest caries treatment costs on average were those born after CWF cessation.

**Conclusions:**

These results expand our understanding of caries epidemiology under CWF cessation conditions and reaffirm that optimal CWF exposure prevents dental decay. These findings can offer fiscal estimates of the cost burden associated with CWF cessation policies and help decision-makers advance oral health, prevent dental caries, and promote equity in oral health outcomes.

## Introduction

While the oral health of most Americans has improved over the last century, it remains a significant unmet health care need for children and structurally marginalized groups [1, 2]. Dental caries continues to be the most common chronic childhood disease, and dental pain is the second most common cause of school absences [1, 3, 4]. Importantly, the burden of negative oral health outcomes is disproportionately borne by vulnerable groups, including those least able to advocate for themselves, such as children, members of non-majority ethnic groups and low-income families [5–7].

From the 1930s through the early part of the twenty-first century, the research community has dedicated time and resources toward producing and reviewing comparison studies of fluoridated versus non-fluoridated communities [8]. Investigating potential associations among optimal community water fluoridation (CWF) and reductions in both pediatric dental caries and adult tooth loss are also important for improving economic, racial, and ethnic disparities in oral health [9–15]. Over the decades, researchers have established a large body of empirical work supporting both the efficacy and safety standards of CWF systems, which are often summarized in major meta reviews [2, 7, 16–19]. In addition to caries prevention, studies have demonstrated the treatment cost savings secondary to CWF. For example, estimates specify that for every $1 spent on oral health preventative measures, such as CFW, taxpayers can save $50 in annual treatment costs for each low-income citizen who relies on state and federal subsidies for dental care [3, 16, 20–24].

The U.S. Centers for Disease Control, along with several independent commissions, conclude that CWF is both a safe and cost-effective method for decreasing dental disease and caries among populations, regardless of age or income [3, 11, 18, 19, 21, 25]. Two large meta-reviews with different inclusion parameters noted that much of the evidence regarding CWF is based on mid- to late-twentieth century studies and cautioned about the applicability of the findings to modern conditions with widely available fluoridated toothpaste and advanced dental technologies [18, 19]. In 2010, the U.S. Department of Health and Human Services convened an interagency panel to review all the available evidence regarding CWF and its potential positive and negative health effects [7]. The panel determined that even after the introduction and widespread availability of fluoridated toothpaste, U.S. populations still benefited from CWF as a result of reductions in tooth decay up to 25% [7, 14, 21]. This panel also found that the only negative health effect associated with optimal CWF, even at higher than recommended levels (2–4 ppm), was severe dental fluorosis [7]. Therefore, considering the concerns regarding dental fluorosis, particularly for children whose teeth are still developing, the U.S. Public Health Service issued a new recommendation that community water programs fluoridate the water supply at 0.7 mg/L versus the previous range of 0.7–1.2 mg/L given the multiple sources of fluoride in the modern context and to balance decay prevention benefits while reducing the risk of dental fluorosis [2, 7]. Recent trends toward CWF discontinuation in public water systems represent an opportunity to evaluate caries epidemiology and caries treatment cost variations under suboptimal CWF conditions [26]. Given the relatively new phenomenon of policy change inspired by CWF cessation advocacy groups, the impact on population health after removing exposure to optimal fluoride levels in public water systems remains understudied [27–29].

The epidemiological impact of CWF discontinuation has been analyzed in only a small number of studies, and the results have been mixed [25–30]. Additionally, whether specific age groups or income levels are more or less at risk for caries development following CWF cessation has not yet been established [25–30]. The first known meta-analysis of CWF cessation reviewed 13 multidisciplinary databases, and the authors noted only 15 research instances in which CWF cessation was the studied intervention [26]. These publications occurred over several decades, from 1956 to 2003, and represented thirteen different countries [26]. Variations among these published works include methodological factors, such as utilization of a comparison group for whom CWF was not ceased, application of a concurrent cross-sectional versus time series approach, and differences in the type of metric utilized – typically full dental records, claims records or open-mouth screening exams (i.e., DMFT scores), and contextual factors, such as the time interval post-cessation, healthcare delivery systems, local socio-economic conditions and consideration of other sources of fluoride in the community (the introduction of fluoride rinse programs, supplements, or available fluoride salts) [26]. This research intends to contribute to the growing CWF cessation evidence base by assessing and quantifying oral health changes secondary to CWF discontinuation among Medicaid-eligible children and adolescents in Juneau, Alaska, using documentation of caries-related procedures from Medicaid dental claims records six years post-CWF cessation.

According to the previous Surgeon General of the United States, as a nation, approximately 75% of the population has access to optimally fluoridated water [31]. The Healthy People 2020 [32] objective on CWF aims to increase that percentage to 79.6% of the population receiving the optimum level of CWF associated with caries prevention (0.7 mg/L or 0.7 ppm) in community water sources. However, the percentage of Alaska’s population served by CWF was 65% in 2007 and fell to 49.3% in 2014 as a result of local city officials changing the public water fluoridation policies [33]. City assemblies in both Juneau (2007) and Fairbanks (2011) reversed CWF policies citing a ‘lack of evidence’ regarding the oral health benefits and risks of CWF [34]. Currently, the oral health impacts of these local water policy decisions on population oral health in Alaska remain unknown.

This research aimed to assess the impacts of these decisions using the following three research objectives. The first objective was to determine the extent to which CWF cessation impacts the frequency of dental caries-related procedures among Medicaid-eligible children and adolescents. The second objective was to determine the extent to which CWF cessation impacts caries severity as measured by caries-related treatment costs among Medicaid-eligible children and adolescents. The third objective was to determine the extent to which CWF cessation impacts caries development rates for specific age cohorts among Medicaid-eligible children and adolescents. Recent trends toward CWF discontinuation from public water systems represent an opportunity to evaluate caries epidemiology and caries treatment cost variations under suboptimal CWF conditions [26].

## Methods

Juneau served as an ideal study population, with 96% of the residents in zip code 99801 serviced by city water [35, 36]. The rugged terrain of Southeast Alaska makes Juneau one of only two U.S. state capitals accessible only by plane or sea. While Juneau lacks connections to major road systems, thus mitigating the risk of confounding from optimal CWF exposure due to in-and-out migration or travel from neighboring counties (also known as the ‘halo’ effect), it maintains all the modern conveniences that one would expect in the third largest Alaskan city – including schools, public transportation, a hospital, multiple clinics and a variety of dental professional offices, as well as the Southeast Alaska Tribal Health Consortium (SEARHC) headquarters. We also note the widespread availability of fluoridated toothpaste before, during, and after the study at retail outlets, as well as the distribution of such toothpaste to patients at dental clinics. The annual residential population characteristics are similar to those that we may observe on an island or in a closed population. For example, the Juneau census reported a population of 31,283 in 2003 and 32,832 in 2012, reflecting a total increase of 1549 over the nine-year period. In other words, a small population increase of 0.006%, or 172 persons, per year occurred during the study period [37, 38].

The target population of this study included children and adolescents between the ages of 0–18 years living in families whose incomes met Medicaid requirements. The eligibility requirements for Alaskans seeking Medicaid includes children up to 18 years old if the family income does not exceed 150–200% of the Federal Poverty Level [39]. Medicaid income limits vary depending on family size. The rationale for this focus was to assess two groups living in the same zip code with similar ages and economic experiences at two points in time, thus mitigating the influence of confounding factors known to influence oral health status, such as parent educational attainment and wide variations in income [40]. Families living in poverty also represent a vulnerable group likely to be affected by CWF cessation policy decisions and the group that is least able to participate in health policy decision-making processes [4, 24, 41]. As the entire study population was sourced from a homogeneous economic group at two different time points, we were able to observe the influences of the independent variable (CWF) on the dependent variables (dental caries procedures and treatment costs) both before and after CWF cessation.

The retrospective comparative research design provided a method for investigating the main effect of CWF removal from community water systems on pediatric and adolescent oral health using Medicaid dental claims billing records. Data from Medicaid dental claims have been utilized in previous research, and the form is standardized by the American Dental Association to specify demographic indicators, exact procedure codes, reimbursement rates and provider service charges [16, 22, 23, 42]. Data were secured from all Medicaid dental claims records submitted during 2003, three years prior to cessation, and 2012, six years post-cessation, for all Medicaid-eligible children aged 0 to 18 years residing in the 99801 zip code who were examined by a dentist. The State of Alaska Chief Dental Officer confirmed that Juneau had been optimally fluoridated since the early 1980s, noting a ‘fluoride stoppage’ during the last half of 2003 to study pipe corrosion, although documentation of the actual study was not available (Dr. Whistler, personal communication, February 2, 2016). Therefore, the year 2003 was selected to obtain a clean representation of optimal (0.7 mg/L-1.2 mg/L) CWF exposure. Medicaid claims records were not available from the central Medicaid processing center for the period after 2012 for Alaska; therefore, as CWF cessation occurred in January of 2007, 2012 was selected as the comparison year to maximize the number of children in the sample with only suboptimal (< 0.065 mg/L) CWF exposure as measured annually from 2007 through 2012 [35].

Walden University’s Institutional Review Board approved the study (Walden IRB #10–31–16-0075333). Then, the dental claims database was released after approval by the Centers for Medicare and Medicaid Research Unit [43, 44]. Due to high database costs ($10,500 USD) and study time constraints, only two years of claims were purchased.

The entire study population, including both 2003 and 2012, yielded 1905 patients, exceeding required sample size estimates. All dental claims records for every individual meeting the zip code and age criteria during the study years were reviewed and coded according to study parameters (i.e., Levels 1–4). In 2003, under optimal CWF conditions, the sample size for the 0- to 18-year age group was 853, and in 2012, under suboptimal CWF conditions, the sample included all claims for 1052 patients. Nationally standardized dental code reference material, specifically, Current Dental Terminology (CDT) codes used for procedure and service claim reimbursement, were publicly available for referencing procedure types and costs for both study years. Overall, CDT claims reimbursement rates do not change year to year like Current Procedural Terminology (CPT) codes typically used in medicine. According to the retired State Dental Chief Dr. Whistler (personal communication August 1, 2018), Medicaid made adjustments in the form of increases for dental claims CDT codes in 2009 and 2010, which may have resulted in Medicaid reimbursement increases even without provision of more services. From 2003 to 2008, the Medicaid dental reimbursement rate would largely have been the same (i.e., remained unchanged). Therefore, since this study spanned 2003 and 2012, provider service fees were the more consistent metric and could also be adjusted for inflation, thus allowing comparisons*.*

Further variables were developed to accurately address the research objectives. In addition to sorting data into age group cohorts, a variable reflecting the number of caries-related procedures and total costs for caries-related procedures was used. More specifically, all dental procedure codes were organized into four levels. Level 1 represented the type of oral exam (e.g., partial or comprehensive), Level 2 represented preventative care (e.g., x-rays, sealants or fluoride varnish), Level 3 represented caries-related services (e.g., restoration by amalgam, resin, crown, filling under sedation or endodontic/root canal treatments), and Level 4 represented all other services, such as extractions and surgeries. While some Level 4 procedures were likely caries-related, such as extractions, we were not able to confirm this assumption by CDT codes alone as they lack diagnostic details. Therefore, to maximize precision regarding decay without a full medical record, the claims for extractions and outpatient surgeries were not included in the analysis.

The study objectives required analysis of the Level 3 category of procedure claims. We manually counted the number of caries-related claims (Level 3 claims) and the total dollar amount charged by the service provider for these restorative treatments. For example, if a patient had a one-surface primary amalgam restoration and a three-surface anterior resin restoration during the study year, then this patient’s experience would be summed as two caries-related procedures, along with the total caries-related costs for these specific procedures.

Descriptive statistics were calculated in SPSS for the independent variable of CWF and the dependent variables of dental caries procedures and dental caries-related costs, followed by adjustments for gender and race. Adjustments for income and parental educational attainment were not applied since the entire sample included only low-income participants whose family incomes met the criteria for Medicaid eligibility. Parent education was not a variable in the database, but it is assumed to be similar between the two study years since the child or adolescent was receiving Medicaid benefits. Qualification for Medicaid was and remains based on income level and varies by family size, disability status, and other metrics. For example, in 2003, the poverty level for a family of three in Alaska was defined as an annual income of $15,140, and in 2012, the income level was $23, 870 [45, 46]. Medicaid expansion in Alaska did not occur until 2015 under the Affordable Care Act. Proximity to a dental provider in the small community of Juneau, which utilizes public transit and has approximately 40 miles of paved, two-lane highway, remained unchanged during the study period.

## Results

Univariate analysis of the data indicated that half of the participants were male (51.2%). Slightly more than one-half (53.9%) of the participants self-identified as American Indian or Alaska Native (AI/AN), and 30.9% self-identified as white/Caucasian. While the AI/AN community accounts for only 13.4% of Alaska’s total population, they are over-represented in the Medicaid group due to historical oppression resulting in poverty. Table 1 summarizes the full descriptive statistics of the complete study population for the 0- to 18-year-old age groups.

**Table 1:** Descriptive Statistics of the Juneau Study Sample by CWF Status (*n* = 1905)

Descriptor	Optimal CWF Year 2003 (0.7–1.2 mg/L)		Suboptimal CWF Year 2012 (< 0.1 mg/L)	
	Frequency	Percent	Frequency	Percent
*N*	853	44.8	1052	55.2
Gender				
Female	402	47.2	528	50.1
Male	451	52.8	524	49.9
Race/Ethnicity				
White/Non-Hispanic	319	37.5	269	25.6
Black/African American	19	0.02	19	0.02
American Indian or Alaskan Native	423	49.6	604	57.4
Asian or Pacific Islander	23	2.7	37	3.5
Hispanic or Latino	18	2.1	52	4.9
Native Hawaiian or Other Pacific	18	2.1	55	5.2
Unknown	33	3.9	16	1.5

We conducted a bivariate analysis of the mean number of caries procedures for the study groups under both conditions to address the first research objective assessing the extent to which CWF cessation impacts the frequency of dental caries procedures. According to the results of a Shapiro-Wilk test (*p* < 0.0001), the data were not normally distributed. Therefore, a Mann-Whitney U test was used to evaluate the difference in the mean number of dental caries-related procedures per child between the two independent CWF groups of different sizes. The results in Table 2 demonstrate that the mean number of caries-related procedures for the 0- to 18-year-old age groups was significantly higher in the suboptimal CWF group than that in the optimal CWF group (2.35 vs. 2.02, *p* < 0.001). The binary logistic regression results indicated that the odds ratio for patients aged 0 to 18 years living under optimal CWF conditions to receive a dental caries procedure was 0.748, indicating a protective effect (OR = 0.748, 95% CI [0.62, 0.90], *p* < 0.0001). In other words, the odds of a child or adolescent undergoing a dental caries procedure in 2003 was 25.2% less than that of a child or adolescent in the suboptimal CWF group.

**Table 2:** The Mean (SD) Number of Caries Procedures per Child in 2003 and 2012, and a Summary of the Bivariate and Binary Regression Analyses

*Age Group (years)*	*Mean (SD)* *2003 Optimal CWF*	*Mean (SD)* *2012 Suboptimal CWF*	*Mann-Whitney U* *p*	*Logistic Regression* ^*a*^ *Optimal CWF OR, [95% CI]*
0 to < 6	1.55 (3.89) *n* = 194	2.52 (4.35) *n* = 301	0.0001	0.488, [0.33, 0.73]
0 to < 7	2.01 (4.22) *n* = 303	2.68 (4.57) *n* = 461	0.004	0.699, [0.52, 0.95]
7 to < 13	1.61 (3.38) *n* = 352	1.64 (2.60) *n* = 400	NS	NS
13 to 18	2.75 (4.73) *n* = 198	3.04 (4.66) *n* = 191	NS	NS
0 to 18	2.02 (4.05) *n* = 853	2.35 (3.99) *n* = 1052	0.001	0.748, [0.62, 0.90]

*NS* Not Significant

^a^Adjusted for gender and race

Our analysis for the second research objective yielded similar results (Table 3). The mean caries-related treatment cost for the 0- to 18-year-old age cohort was significantly higher in the suboptimal CWF group than that in the optimal CWF group ($593.70 vs. $344.34, *p* < 0.0001) without adjusting for inflation. According to the U.S. Department of Labor Consumer Price Index [47], the inflation rate increased an estimated 24.75% between 2003 and 2012. Therefore, the increase in inflation-adjusted provider service charges in caries treatment costs associated with CWF cessation for the 0- to 18-year-old age group was + 47%, or $161.84. According to logistic regression analysis, the odds that a patient aged 0 to 18 years under optimally fluoridated conditions would be billed for dental caries treatment was 0.749, which was 25.1% less than that for a patient of the same age in the suboptimal CWF conditions group (OR = 0.749, 95% CI [0.623, 0.90], *p* < 0.002).

**Table 3:** Mean Caries-related Treatment Costs by Age in 2003 and 2012 and Adjusted for Inflation

*Age Group* *(years)*	*Mean ($)* *2003 Optimal CWF*	*Mean ($)* *2012 Suboptimal CWF*	*Mann-Whitney U* *p*	*Total Cost Inc/ % Inc*	*Adjusted* ^*a*^ *−25% Inflation*	*Increase ($) Attributed to Suboptimal CWF*
0 to < 6	272.72	644.72	0.0001	372.00/136%	111%	302.71
0 to < 7	350.13	692.87	0.0001	342.74/98%	73%	255.60
7 to < 13	241.52	382.44	0.001	140.92/58%	33%	79.70
13 to 18	519.07	795.68	0.035	276.61/53%	28%	145.34
0 to 18	344.34	593.70	0.0001	249.36/72%	47%	161.84

^a^Service provider charges were used rather than Medicaid reimbursement amounts for comparisons by accurately adjusting for inflation

Lastly, our analysis for the third research objective considered which age group suffered the largest caries procedure burden under both CWF conditions. Bivariate analysis revealed that the mean number of caries-related procedures per patient for children under 7 years old was significantly higher in the suboptimal CWF group than that in the optimal CWF group (2.68 vs. 2.01, *p* < 0.004). The results from binary logistic regression were also significant (OR = 0.699, 95% CI [0.52, 0.95], *p* < 0.02) and similar to previous results, indicating a protective effect for optimal CWF exposure. Caries treatment costs were also higher in the under 7-year-old suboptimal CWF group than those in the optimal CWF group ($692.87 vs. $350.13, *p* < 0.0001). After adjusting for inflation, we observed a caries treatment cost increase of 73% attributable to CWF cessation and estimated at approximately $255.60.

**Fig. 1:**
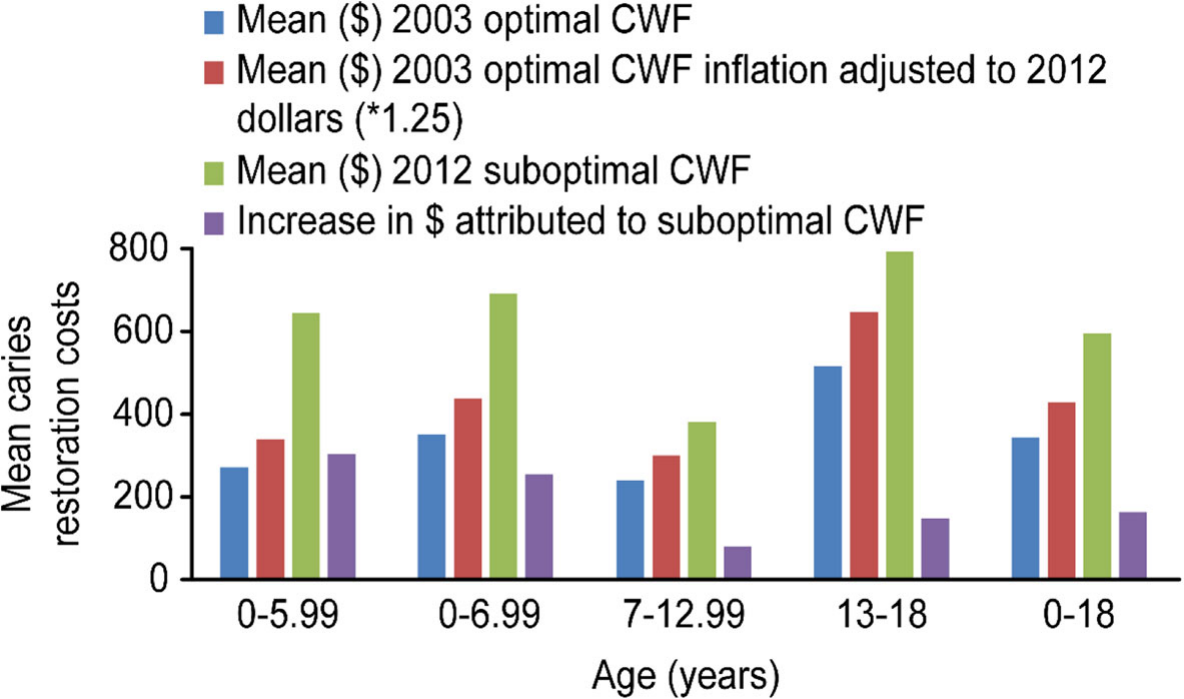
Mean caries restoration costs according to age for children with optimal or suboptimal community water fluoridation (CWF). First, the mean cost in 2003 for children with optimal CWF was calculated; the mean cost in 2003 was then adjusted for inflation (*1.25) according to the 2012 dollar value; and lastly the increase cost attributed to suboptimal CWF was calculated

The results for the group of individuals born after CWF cessation and aged < 6 years were of particular interest to the research team. These results are also included in Tables 2 and 3 and Fig. 1, which summarize the differences in the mean number of caries procedures and treatment cost results and illustrates consistent epidemiologic trends. The children without exposure to optimal CWF suffered a higher percent increase in caries procedures along with higher restoration costs, thus signifying greater tooth surface loss to decay secondary to weaker enamel (2.52 vs. 1.55, *p* < 0.0001) ($644.72 vs. $272.73, *p* < 0.0001) (Fig. 1). According to binary regression analysis for the under six-years-old age group, the odds of undergoing dental caries procedures under optimal CWF conditions was 51% less than that for a child of the same age in 2012 under suboptimal conditions (OR = 0.488, 95% CI [0.33, 0.73], *p* < 0.0001).

## Discussion

The results of this study confirm the benefits of optimal CWF supported by previous research and can contribute additional research-based evidence regarding the oral health consequences and costs that can arise when CWF is discontinued. The most common modality of study among previous CWF cessation research was a concurrent cross-sectional analysis using DMFT screening in a community that had ceased CWF at some point in the past compared to a community that continued optimal CWF [26]. Therefore, this research offers an alternative modality for studying the effects of CWF using Medicaid claims data from the same community before and after CWF cessation.

The results demonstrate a statistically significant increase in the number of dental caries procedures and associated treatment costs for the general cohort under suboptimal CWF conditions in 2012 among patients aged 0 to 18 years (2.35 vs. 2.02, *p* < 0.001). This increase was more pronounced among younger age groups under suboptimal CWF conditions, specifically the < 7-year-old age group (2.68 vs. 2.01, *p* = 0.004) and the < 6-year-old age group who were born after CWF was ceased (2.52 vs. 1.55, *p* < 0.0001).

The naturally occurring fluoride level in Juneau’s water since CWF was ceased in January 2007 has remained between 0.05–0.065 mg/L per annual reporting, which is more than ten-times less than the optimal CWF level needed for caries prevention [35]. Previous research indicates that without the presence of optimal levels of fluoride in drinking water, and thus in the mouth and saliva, teeth may form with weaker enamel and lack the ability to remineralize early signs of decay [9, 25, 31, 48]. Therefore, we expected to observe a general increase in dental caries-related procedures and treatment costs across age groups because fluoride acts primarily topically [9, 48]. Additionally, we expected to observe more significant impacts among patients with the lowest exposure to optimal CWF.

In this study, a major advantage of acquiring both pre- and post-fluoride cessation data in this natural setting was the potential to assess the net differences in the intervention condition (suboptimal CWF) and in the control condition (optimal CWF) [49]. The results indicate a clear caries epidemiologic shift toward a caries increase among patients without the protective benefit of optimal CWF. As no significant difference in the mean number of caries procedures was observed between the 7- to 12- and 13- to 18-year-old age groups, we suggest that these individuals may have a residual protective effect from optimal CWF exposure during childhood and early adolescence. Additionally, between 2003 and 2012, we observed a sevenfold overall increase in the placement of sealants. Optimal CWF exposure during childhood, plus the increased use of dental sealants during school-aged years, may explain the results among the older cohorts; however, this topic requires further investigation [27–29].

Overall, we were able to measure the influence of the independent variable, CWF, on the dependent variables, dental caries procedures and treatment costs, both before and after cessation by utilizing a study population comprising participants living at or near poverty conditions. The similarity of the target groups’ income criteria for Medicaid eligibility adds strength to the internal validity of the study; therefore, the results and causal implications are more valid than if the results had been derived from the general population [22, 49]. Second, the external validity of the results was also increased by working with only Medicaid claims data, which limited the influence of higher-income groups [22, 50]. Families with high incomes may have easier access to dental care and may more routinely visit a dentist or refill supplemental fluoride tablets, which could potentially dilute small changes in caries rates under fluoridation and non-fluoridation conditions [22, 24, 27, 50]. Therefore, the results are likely generalizable to other Medicaid groups in communities considering CWF cessation.

Regarding reliability, variations in providers’ therapeutic approaches and billing practices may have had some influence, but we anticipate that these variations were similar in both study years. The data were managed and recoded by only two individuals, and errors were estimated to be minimal. In summary, the internal and external qualities of this study support generalizability to other 0- to 18-year-old Medicaid populations in Alaska who have already ceased CWF or are considering CWF cessation. The methodology and analysis processes are certainly transferrable to other regions and offer an innovative metric option for future oral health research and statewide public health surveillance programs.

The results presented in Table 3 indicate a progressively higher caries risk and cost burden for the younger age groups in the suboptimal CWF cohort. Please recall that older patients in this study were exposed to several years of optimal CWF as young children born prior to cessation in 2007. For example, patients in the 7- to 13-year-old age group were born between 2000 and 2005 and thus benefited from early-life/childhood optimal CWF exposure. Overall, the costs of caries treatment services increased for each age group cohort, even after adjusting for inflation, and were markedly higher under suboptimal CWF conditions. These results support current evidence that even in modern conditions with widely available fluoride toothpaste, rinses, and professionally applied prophylaxis, CWF is associated with population benefits, including cost effectiveness and caries prevention [6, 7, 22, 24, 31, 50, 51].

### Limitations

The study has a number of limitations. First, due to funding limits, only two years of data were purchased for comparison rather than five to ten years of data, which would have enabled a more sophisticated trend analysis. Second, dental claims for extractions or full-mouth reconstruction were removed from the primary data analysis because we could not confirm that these procedures were caries-related without the clients’ full medical records. Therefore, caries procedures and costs may be underrepresented in the results. Third, the coding scheme also assumes that within 1 year, the dental professional treated all points of decay for each individual patient and did not over- or undertreat. Lastly, if an eligible child did not visit the dentist that year, then no claims forms were generated, and they were not included in the study.

While the use of reimbursement claims records as the primary metric for longitudinal evaluation in a pre- and post-CWF-exposed population was innovative, a precedent had been set by concurrent comparison population analyses completed in Louisiana [16] and New York [22] and by the Texas Department of Public Health [23]. Additional methodological factors include identification of strategies to control for confounders and the issue of measuring short-term versus long-term changes. Rugg-Gunn and Do [40] remark that among studies published in the last twenty-five years exploring CWF using a cross-sectional comparison methodology, the use of multivariate statistical analyses with adjustments for confounders has yielded minimal change in the net effect of CWF on caries reduction. Typical covariates for dental caries and dozens of other negative health outcomes include diet, parental education and parental income [40]. As with most negative health outcomes, these covariates can play roles in determining an individual’s oral health, often influencing diet options, social norms toward seeking preventative dental care, prioritization of home oral care practices and stress levels [24, 28]. We acknowledge baseline and comparison data regarding parental education among low income families, home oral hygiene practices, and dietary habits to be unknown considerations. However, proxy measures may offer some estimates of influence. For example, the Alaska Youth Risk Behavioral Survey (YRBS) tracks sugar-sweetened beverage (SSB) consumption among teens, which appears to be declining, mirroring national trends. According to the YRBS for Alaska in 2007, 21.8% of youths drank one SSB one or more times per day during the past 7 days, while in 2013, the rate was 15.8% [52, 53]. While the trend of reduced SSB and thus added sugar consumption may be declining nationally and statewide, the reduction is highly unlikely to be sufficient to result in a reduction in caries (< 10% of total daily calories) [54, 55].

Based on available population estimates, we note only small changes between the 2003 and 2012 Juneau Borough censuses and minimal changes to Medicaid eligibility requirements. Therefore, common sense would support that the parental education level among Medicaid-eligible families was also comparable between the two groups. While the similarities in population size and socioeconomic conditions may strengthen the validity of the study conclusions, they can also be viewed as a limitation for the generalizability of the study’s results to other populations that experience more in-and-out migration or wider variations in income.

Other covariates that may have influenced the results include prescriptive fluoride supplementation, school fluoride rinse programs, and dental sealants. No school-based oral health or school rinse programs existed in Juneau before, during or after the study period, and prescriptive supplementation has always remained very limited (personal communications with Dr. Whistler and Dr. Hort, January 2016). Notably, among studies exploring other probable confounders, such as widely available fluoride toothpaste, moderate access to school-based fluoride varnish programs and in-office fluoride applications, researchers have reported that optimal CWF still improves oral health among children through caries prevention, enamel remineralization and cost savings [22, 28, 31, 50, 51, 56, 57]. Lastly, another potential limitation of this study is that we do not have information regarding fluoride toothpaste use in this low-socioeconomic status population, and fluoride toothpaste use may be much lower than that expected in other populations. Such information could strengthen the conclusion regarding the benefit of CWF in this population compared to other populations that regularly use fluoride toothpaste.

## Conclusion

This study analyzed oral health changes secondary to CWF discontinuation among Medicaid-eligible children and adolescents in a community in which the local government ceased fluoridation of the public water system. We examined the relationship between dental caries-related procedures and costs under optimal and suboptimal CWF conditions through rigorous statistical analysis of Medicaid dental claims records and formed the following conclusions. According to the aforementioned results, CWF cessation promoted a marked increase in the number of caries-related procedures and treatment costs for Medicaid-eligible children and adolescents aged 0–18 years. Additionally, the results indicated that children in the younger age group cohorts underwent more dental caries procedures than the older age group cohorts, who had benefited from early childhood exposure to optimal CWF. These results add to the growing body of information available regarding CWF cessation epidemiology by both confirming the dental caries prevention benefit of CWF and expanding the evidence base regarding the oral health impacts of CWF cessation under contemporary conditions.

The analysis also can offer fiscal estimates that can be used by community leaders and decision-makers who are considering CWF cessation and may need to plan for the increased revenue required to address the treatment costs among clients relying on state and federal government subsidies. With this study, dental and public health professionals also have access to more evidence to accurately inform officials establishing future community water fluoridation policies and to illustrate how CWF cessation can affect individuals, especially children, in economically vulnerable or low-income circumstances [22, 24, 28, 50].
